# Bent crystals for efficient beam steering of multi TeV-particle beams

**DOI:** 10.1140/epjc/s10052-018-6196-z

**Published:** 2018-09-06

**Authors:** A. Mazzolari, M. Romagnoni, R. Camattari, E. Bagli, L. Bandiera, G. Germogli, V. Guidi, G. Cavoto

**Affiliations:** 1 INFN Sezione di Ferrara, Via Saragat 1, 44122 Ferrara, Italy; 2 Dipartimento di Fisica e Scienze della Terra, Università degli Studi di Ferrara Via Saragat 1, 44122 Ferrara, Italy; 3 Sapienza Università degli Studi di Roma and INFN Sezione di Roma, Piazzale Aldo Moro 2, 00185 Rome, Italy

## Abstract

Charged particle beams can be manipulated by exploiting the channeling phenomenon in bent crystals. Two plate-like crystals, bent by mechanical holders, were manufactured and characterised for such purpose at the Sensor and Semiconductor Laboratory in Ferrara, Italy. An anticlastic curvature was obtained for these crystals, achieving a steering angle of the order of 1 mrad, which is about 20 times larger than the values currently achieved for the bent crystals used in the LHC for collimation experiments. Finally, a Geant4 simulation was performed to study the channeling efficiency for beam deflection with 400 GeV/c and 7 TeV/c proton beams. Such crystals represent technological progress in the development of bent crystals for highly energetic charged particle beams. Indeed, they are designed to impart an angular kick to a 7 TeV/c proton beam with unprecedented high efficiency. Therefore, this study demonstrates the possibility of realizing bent crystals suitable for beam extraction in high-energy hadron accelerators, such as LHC or at the future FCC. A further series of studies should be conducted to evaluate the channeling efficiency and the deflection angle of the realized crystals via a charged proton beam.

## Introduction

Modern particle accelerators, such as the Large Hadron Collider (LHC) at CERN, offer unique opportunities to explore the nature of fundamental interactions of matter. They deliver colliding beams of particles at the highest energy ever achieved with an unprecedented intensity. The possibility of the extraction of a multi-TeV beam for fixed target experiments is becoming more and more appealing in the high-energy physics community. Both the energy and intensity frontiers need cutting edge technologies and new ground-breaking ideas in the context of both beam manipulation and particle detection. Concerning the former, the possibility of extracting from the LHC its multi-TeV hadron beam for fixed target experiments could be important for the high-energy physics community. Such an extraction line would open a new domain for the investigation of the strong interactions in the negative rapidity range, offering new tests of Quantum Chromo-Dynamics and with an interesting application to the study of cross section of TeV-hadrons with light elements | important to understand the showers of the Ultra High Energy Cosmic Rays [1]. Another remarkable use of an extracted beam from the LHC would be the measurement of magnetic dipole moments of short-lived heavy-quark baryons exploiting the spin precession occurring for a channeled particle along a bent crystal [2]. More interestingly, this idea can be exploited in the search for electric dipole moments of strange and charm baryons, thus opening a novel way of new physics investigation [3, 4].

A solution for the extraction of the multi-TeV proton or heavy ion beams of the LHC could be a non-resonant scheme in which the beam halo is populated introducing electromagnetic noise in the machine [5]. Indeed, lateral diffusion of the beam particles can be artificially enhanced by using asynchronous electromagnetic noise injection. Then, the beam halo can be deflected through a bent crystal out of the beam pipe towards a target, exactly as a kicker magnet would do.

This is possible since the charged particles that impinge at small angles with respect to the lattice planes perceive the regular electric field of the crystal. As a result, the particles undergo the channeling effect crossing the whole crystal. If the crystal is bent, the particles follow the crystal curvature and exit the crystal being deflected from their original trajectory [6, 7]. Indeed, owing to this intense electric field, a few mm long bent crystal is capable of producing a beam deflection equivalent to that of a magnetic field larger than $10^3$ Tesla. A halo extraction rate of $10^9$ particles per second would be very interesting for fixed target experiments with 7 TeV protons, calling for a crystal extraction efficiency of about 50% [8, 9].

## Extraction of a charged particle beam - brief excursus

Beam extraction from particle accelerators is typically accomplished by means of magnetic devices. Alternatives based on the use of crystals have been successfully investigated worldwide.

The first attempt to extract a circulating beam from an accelerator by means of a bent crystal was performed at Dubna Synchrophasotron [10], where an 11 mm long crystal operating in parasitic mode diverted a 8.4 GeV proton beam at an angle of 35 mrad with an efficiency of $\sim 10^{-4}$. Later, an intensive experimental campaign was carried out at U-70 (IHEP, Protvino, Moscow), where 50 and 70 GeV proton beams [11, 12] were extracted with an efficiency of about 1%. The low extraction efficiencies were ascribed to sub-optimal geometries of the crystals [6] or to a non-optimized experiment set-up [10]. Indeed, such a large deflection angle led to a very low channeling efficiency. Moreover, the curvature of such crystals was highly inhomogeneous, causing further dechanneling of the particles.

Although the obtained results showed low efficiency, they demonstrated for the first time the channeling phenomenon. Afterwards, an experimental campaign was started in the late 1990s at CERN (proton beams of energies of 14, 120 and 270 GeV [13–15]) and Tevatron (proton beam of energy 900 GeV [16, 17]). Extraction efficiencies increased ($\sim 10\%$ and $\sim 25\%$ respectively), but still remained at values consistently lower than the ones reached with slow extraction approaches $(\sim 98\%)$. Such low values were ascribed to the presence of an imperfect layer on the crystal surface [14, 18, 19], unwanted parasitic effects in the deformation of the crystal [13, 19], and to an inappropriate choice of the length of the crystal [8, 18, 20, 21]. In particular, simulations demonstrated [18, 19] that the length of the crystals used at SPS and Tevatron (30 and 40 mm respectively) was optimized for operations in single-pass mode, not in multi-pass. As a result, crystals about 5 times shorter would have provided extraction efficiencies about 3 times higher. Moreover, recent simulations predict the possibility to reach an efficiency of 99% for 270 GeV proton extraction from the SPS [22].

Simulation models proved to be invaluable tools in the design of crystalline deflectors and drove the design and manufacturing of a subsequent generation of crystals [23] and bending schemes [24, 25]. This progress resulted in a considerable increase of extraction efficiency, which reached the value of 85% [24].

Further developments, carried out in the last 20 years in crystal manufacturing and characterization, allowed to manufacture crystals free from lattice damage, surface roughness lower than 0.1 nm, and miscut angle lower than $5\,\upmu \hbox {rad}$, satisfying the requirements to aim at the collimation or the extraction of the LHC circulating beam. Driven by those results, an intense experimental campaign [26] investigating the possibility to collimate or extract the proton beam circulating in the LHC was started in the late 1990 at H8 and H4 extracted lines of the SPS. New coherent interaction effects of ultra-relativistic proton beams with crystals were observed, among which were the phenomena of volume reflection [27, 28], beam steering by means of crystal axes [29–31], multiple volume reflection [32–35] and mirroring [36]. For the first time, efficient beam steering was also recorded for both positively [37–39] and negatively charged particle beams [40, 41].

The success of this campaign led to the use of the SPS as a platform for developing the technology needed to attempt the collimation of the LHC circulating beam [32, 42, 43]. Successively, bent crystals were installed in the LHC [44], where coherent interactions even at 6.5 TeV were observed [45]. This result opened the possibility to achieve crystal-assisted collimation of the LHC circulating beam. Despite the good results achieved, the features of the crystals used for the channeling experiment at sub-TeV energy have to be adjusted to work efficiently in the multi-TeV energy domain. Therefore, a new generation of bent crystals has been designed to obtain an extracted line from the LHC. In particular, shape and radius of curvature of the crystals for LHC have to be appropriately modified, as will be explained below.

In this paper, we propose to use bent plate crystals for the extraction of multi-GeV and multi-TeV particle beams, i.e. samples with two sides much larger than their thickness. Indeed, up to now only strip-like samples have been produced and tested, i.e. samples with only one dimension larger with respect to the other two. In particular, here two plate crystals have been manufactured, bent, and characterised through interferometric profilometry and through X-ray diffraction. Finally, simulations to estimate the channeling efficiency for the manufactured crystals are shown.

## Extraction of the LHC beam - the CRYSBEAM project

While the crystal collimation scheme requires an angular kick of a few tens of $\upmu \hbox {rad}$ by keeping the halo particles within the beam pipe radius, beam extraction out of the beam pipe would require a deflection angle of $\phi \sim 1$ mrad. This approximate value of the deflection angle was found to be appropriate in a conceptual scheme of extraction in the LHC [46]. In fact, the aim of collimation is to deflect the beam towards an absorber located in the beam pipe downstream of the crystal. On the other hand, a large angle is required for beam deflection to achieve an adequate separation of the extracted beam from the primary beam. In order to attain such a large deflection, it is possible to either increase the thickness of the crystal along the beam, i.e. its length (*L*), or decrease the radius of curvature (*R*), since the deflection is $\phi =L/R$. However, it is not possible to arbitrarily decrease *R*, as it would negatively affect the channeling efficiency [39]. In particular, the deflection efficiency is relatively high only when $R > 6R_c$ [39], where $R_c$ is the critical radius of curvature. For the case of a 7-TeV/c proton beam, $R_c \sim 12$ m [45]. Since the channeling efficiency for a beam of positive particles at 7 TeV/c is weakly affected by the crystal length, a hundred mm long crystal would in principle achieve a high channeling efficiency, of the order of 80–90%. Thus, the optimal configuration for extracting the LHC beam would be a crystal featuring *L* $\sim 100$ mm and *R* $\sim$ 100 m.

**Fig. 1:**
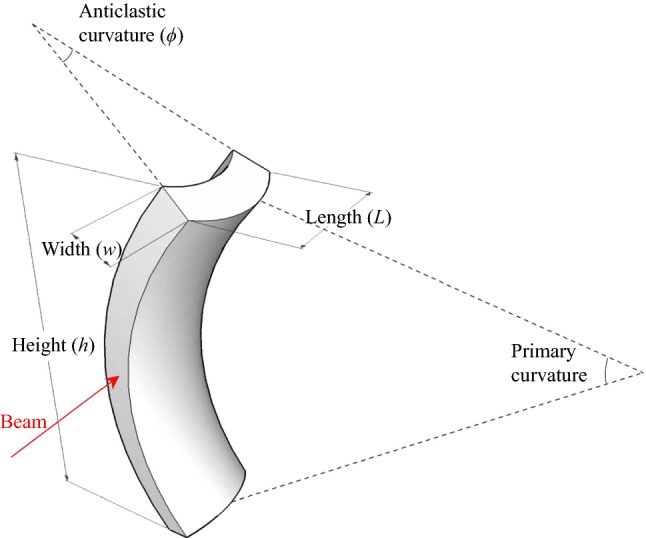
Schematic representation of a crystal with anticlastic deformation. The beam is deflected by the anticlastic curvature, which is perpendicular to the primary bending

The crystals currently used at LHC for collimation tests are made of silicon. In order to deflect charged particle beams, anticlastic [24, 25, 44] or quasi-mosaic [47, 48] deformation can be used. Unfortunately, the quasi-mosaic effect does not allow to operate with a $\sim$100 mm long crystal. Indeed, the quasi-mosaic effect consists in the bending of a family of lattice planes which are perpendicular to the surface of the primary curvature. Thus, in the case of a plate crystal, its thickness would be of the order of $\sim$100 mm, i.e. a very large sample, impossible to bend. On the other hand, the anticlastic deformation can be exploited to realize crystals whose length along the beam direction can be even hundreds of mm. We briefly recall that the anticlastic deformation arises as a consequence of bending a bar along one of its main direction and manifests as a bending of opposite sign along the perpendicular direction (see Fig. 1).

**Table 1:** Parameters of the samples

	Sample 1	Sample 2
Channeling plane	(110)	(111)
Channeling axis	$<100>$	$<110>$
Height (*h*) - x [mm]	$55.0\pm 0.1$	$39.0\pm 0.1$
Length (*L*) - y [mm]	$23.8\pm 0.1$	$56.8\pm 0.1$
Width (*w*) - z [mm]	$0.52\pm 0.02$	$1.00\pm 0.02$
Density of dislocations [1/$\hbox {cm}^2$]	$<1$	$<1$
Interferometric measurement results		
Bending angle $\phi$ ($\upmu$rad)	$1877\pm 94$	$716 \pm 36$
Anticlastic *R* (m)	$12.1 \pm 0.6$	$79.9\pm 4.0$
XRD measurement results		
Bending angle $\phi$ ($\upmu$rad)	$1840\pm 24$	$714\pm 24$
Anticlastic *R* (m)	$12.9 \pm 0.6$	$79.6\pm 4.2$

Up to now, the manufactured crystals for beam deflection feature a strip-like shape, i.e. the crystal length (*L*) is much smaller than its height (*h*). As an example, the crystals exploiting the anticlastic deformation and currently installed it the LHC are 4 mm long (*L*) and 39 mm high (*h*), resulting in an aspect ratio $L/h \simeq 0.1$. For such crystals, the primary deformation was achieved by imposing a bending moment to the crystal through an *ad-hoc* crystal holder. In particular, such strip-like crystals were clamped at their edges to the holder, while the whole structure of the holder was bent through a system of fine screws, causing the controlled deformation of the samples.

Unfortunately, the geometry developed for the crystal collimation cannot be rescaled for beam extraction because a crystal with length along the beam of $\sim$ 100 mm and the same aspect ratio would result in a 1 m high crystal. Even without considering the need for an appropriate bending device, it would be too large and bulky for an installation in a beam pipe.

In this paper, we propose to keep *h* at about 40 mm and to increase *L* to about 100 mm, i.e. a crystal with an aspect ratio of 2.5. Such crystals are under development in the context of the CRYSBEAM project [49]. For such new crystals, the usage of the described bending scheme would strongly suppress the anticlastic deformation [50, 51]. Thus, we manufactured a new bending device that is a redesign of the bending device previously developed. In particular, this new holder allows us to apply two identical and adjustable moments at two crystal edges of a plate crystal and to enhance its anticlastic deformation, instead of suppressing it; more details on this bending device will be reported in a dedicated paper.

## Crystal manufacturing and bending

**Fig. 2:**
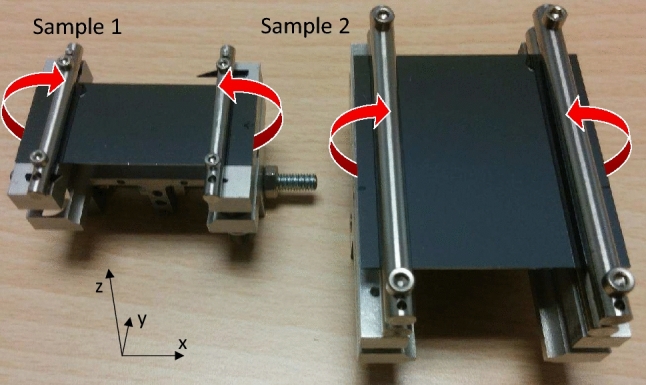
Picture of the samples. The red arrows represent the imposed primary curvature

Two different samples were produced and characterised at the Sensor and Semiconductor Laboratory of the University in Ferrara (SSL), namely sample 1 and sample 2. The samples are made of high quality silicon, with a density of dislocation $<1/\hbox {cm}^2$, as certified by the manufacturer. Sample 1 was produced with $L/h <1$. It was mounted on a holder similar to the one used for the strip-like samples. On the other hand, sample 2 was manufactured to feature $L/h >1$. This sample is the first one with these characteristics that is bent by means of a holder for such a purpose. All the sample parameters are listed in Table 1. A photo of the samples is shown in Fig. 2.

Sample 1 and sample 2 were manufactured to be $520\pm 20\,\upmu$m and $1000\pm 20\,\upmu$m wide, respectively, through a merging of the techniques described in [23] and in [52]. First, the two wafers were coated with a 100 nm thick film of silicon nitride $(Si_3N_4)$ via Low-Pressure Chemical Vapor Deposition (LPCVD). Then, the samples were shaped using an automatic diamond blade dicing saw (Disco DAD 3220). This device can perform a high precision straight cut, with an error of $1 \,\upmu$m for a linear translation and of $10^{-2}$ degrees for rotation. Since the cutting process by the dicing saw damaged the material for a thickness of $\sim 10\,\upmu$m along the line of the cut [23, 53], superficial strains and deformations may appear in the sample [54]. Indeed, the dicing process used to shape the crystals causes a damaged superficial layer that is characterized by a large amount of dislocations – about $10^6/\hbox {cm}^2$. Such defects are highly detrimental for the channeling phenomenon, as they can generate a deformation in the crystal that can extend to relatively large distances from the point where the dislocation is generated. Moreover, since the beam would be perpendicular to the damaged surfaces and would pass through them, the particle trajectories would be affected by multiple Coulomb scattering, which eventually leads to a lowering of the channeling efficiency. In order to have a large channeling efficiency of a 7 TeV energy beam, it is estimated that the maximum number of dislocations should not be larger than $1/\hbox {cm}^2$ [55]. Thus, an etching process to remove the part of the crystal rich of defects was performed. The used etching process has been verified to achieve the required dislocation density in [23]. In particular, Rutherford backscattering in channeling condition was used for the purpose, which is a technique to precisely investigate the crystalline quality of the first layers.

In particular, an isotropic etching was performed using a solution composed of 3 parts of *HF* (50%), 5 parts of $HNO_3$ (70%) and 3 parts $CH_3COOH$ (99%). This chemical solution is characterised by an etching rate of $\sim 2\upmu$m/min. A $\sim$100–120 $\upmu$m thick layer was removed from the cut surfaces. The $Si_3N_4$ layer was used to protect the rest of the samples during this process. Afterwards, it was removed by a chemical etch based of *HF* because it could introduce multiple scattering of the proton beam and it would be a contaminant for the pipe of LHC.

**Fig. 3:**
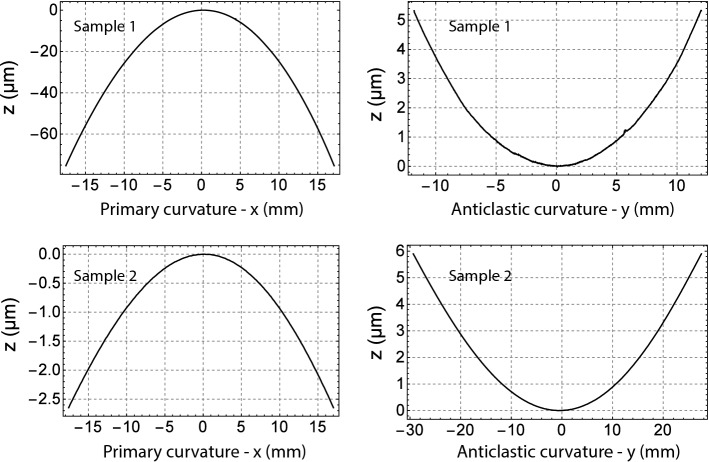
Interferometric measurement of the plate crystals. Left column: deformation along the direction perpendicular to particle beam propagation, i.e. the primary curvature – z vs x position. Right column: deformation along the direction of particle beam propagation, i.e. the anticlastic curvature – z vs y position

**Fig. 4:**
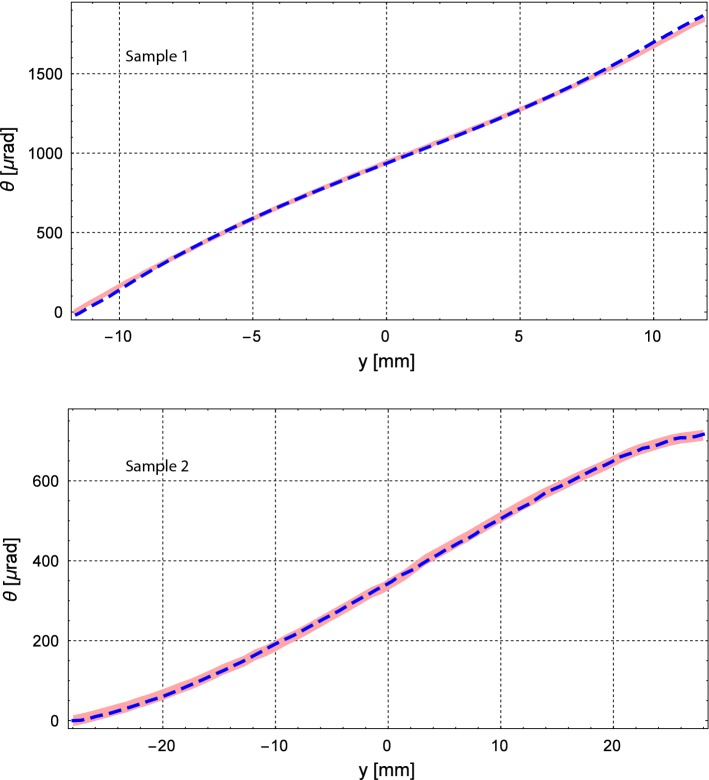
X-ray measurement of the anticlastic deformation for the plate crystals. The red stripes represent the peak values $\theta$ with the instrument uncertainty. The dashed lines represent the numerical derivative of the corresponding interferometric measurements

The crystallographic orientation of the main surface of sample 1, i.e. the channeling planes, was chosen to be (110). Sample 2 was designed to have a larger aspect ratio with respect to sample 1. For this sample, (111) plane was selected as the main face.

## Sample characterization

Two different characterizations were performed to measure the deformation of the manufactured samples. The first characterization was a morphological analysis of the sample surface. It was performed using an interferometric microscope (Veeco NT-1100). This instrument allowed to achieve extreme precision on the vertical axis, up to 1 nm, and has a micrometric lateral resolution.

The morphological characterization of the samples was done before and after the bending process. By comparing the two measurements, it was possible to obtain the displacement induced by the holder, i.e. the curvature of the sample.

The results of the interferometric measurements are shown in Fig. 3. In particular, the deformation at $y=0$ is shown in the left column and corresponds to the primary curvature as measured along a line in the centre of the sample|the origin of the reference system is taken at the centre of the sample. The deformation at $x=0$ is shown in the right column and corresponds to the anticlastic curvature as measured along a line in the centre of the sample, perpendicular to the previous line. The samples show a saddle-like shape, i.e. the sign of the primary curvature is opposite to the sign of the anticlastic curvature.

A numerical derivative has been obtained from the data shown in Fig. 3. These derivatives were used for evaluating the average *R* and $\phi$, which are listed in Table 1. The errors are due to the uncertainty of the stitching process of the interferometric microscope.

In order to directly characterize the deformation of the crystalline planes, the samples were analysed via a high-resolution X-ray diffractometer (HRXRD) PANalytical - X’Pert$^3$ MRD (XL). The analysis was carried out with a monochromatic beam of 8.047 keV (Cu $K\alpha _1$). The angular position $\theta$ of the diffraction peaks was measured with a 3.5 $\upmu$rad precision for various adjacent positions along the direction where the anticlastic curvature occurs. The angular shifts of the peaks correspond to the variation of the lattice planes orientation along the sample width, i.e. the angular deflection $\phi$ that a particle channeled inside the bent crystal would undergo. For these measurements, only the anticlastic deformation was analyzed.

Results of the X-ray characterizations are shown in Fig. 4. The measurement results with the corresponding errors are listed in Table 1.

It is worth noting that the two characterizations are compatible within the errors. To underline the compatibility, the numerical derivatives of the two interferometric measurements have been displayed as dashed lines in Fig. 4. As can be noticed, the dashed lines are in good agreement with the X-ray measurements.

## Simulations

In order to predict the deflection efficiency of the channeling process in bent crystals, Monte Carlo simulations were carried out using the Geant4 toolkit [56, 57]. The Geant4 channeling package [58] with the addition of the DYNECHARM++ [59] and ECHARM [60] codes was used. DYNECHARM++ makes use of the continuum potential approximation proposed by Lindhard [61] and allows the tracking of a relativistic charged particle inside a crystalline medium via the numerical integration of the classical equations of motion. The code has been validated through experiments performed at SPS with 400 GeV/c protons, as in [36, 39, 62]. The computations of the electrical characteristics of the crystals are carried out via ECHARM. The Geant4 application was developed on top of the 10.3 version of the toolkit, which includes the description of crystalline structures [63]. All physical phenomena occurring for a channeled particle are strongly affected by the number of nuclei and electrons encountered, which depends on the particle trajectory [61]. Therefore, the probability of a physics process to occur in the simulation has been weighted as a function of the density of material experienced by a channeled particle. Such dependence of the probability of interaction allows the correct evaluation of the deflection efficiency, which strongly depends on the particle charge sign [41]. The comparison of the simulation produced with Geant4 and experimental data can be found in the literature for planar [64] and axial [29] channeling, proving to be in good agreement with the respective experimental data and also with the analytical calculations.

**Fig. 5:**
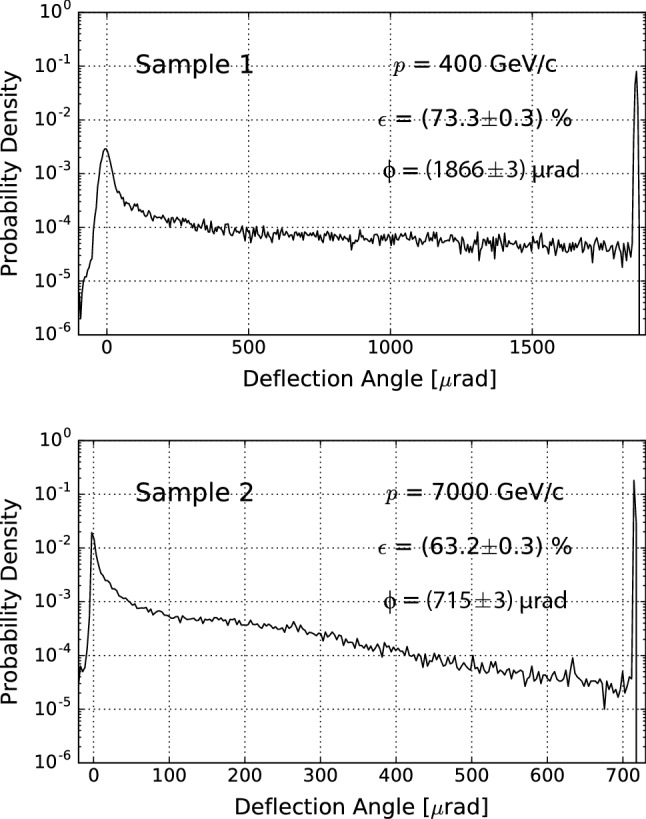
Probability distribution of the deflection efficiency as a function of the deflection angle after the interaction with the crystals. The proton momentum (*p*), the channeling deflection efficiency ($\epsilon$), and the mean deflection angle ($\phi$) are indicated on the figure

The simulation of the interaction in single-pass mode of a collimated beam impinging on the crystals was carried out. In the simulation, a perfectly collimated beam, i.e. with zero divergence, impinged parallel to the main channeling planes. Sample 2 was designed to operate inside the LHC, therefore a 7 TeV/c proton beam was simulated. On the other hand, sample 1 was designed to operate at the SPS energies, thus a 400 GeV/c beam was simulated for this crystal. Figure 5 shows the angular distribution of the particles that exit the crystal after having interacted with it. In the simulation, the crystals were simulated without defects and a negligible experimental uncertainty on the deflection angle is assumed. Sample 1 showed a deflection efficiency under channeling equal to $73.3\pm 0.3\%$, while sample 2 showed a deflection efficiency equal to $63.2\pm 0.3\%$. The sample deformation was simulated by using the data from the XRD measurements (from Fig. 4), considering thus the variation of the curvature radius along the crystal length. The same simulations were worked out also considering a uniform bending curvature (not shown in the figure). For this case, the channeling efficiencies pass from 73.3% and 63.2% to 73.4% and 66.0%, respectively. Such small differences mean that the curvature of the samples was very homogeneous, and the curvature variations weakly affected the channeling efficiency.

## Conclusions

The extraction of the multi-TeV LHC beam for fixed target experiments can be achieved in the near future thanks to the channeling effect in bent crystals. In order to efficiently steer charged particles via channeling, a novel generation of cm-long crystals is needed. An appropriate choice of the bending radius and the absence of crystalline defects are the key ingredients for the fabrication of a crystal capable of efficient deflection of the multi-GeV SPS beam and the multi-TeV LHC beam. Indeed, the need for a crystal free from dislocation is especially relevant for the LHC. In particular, it is important that the crystal is free from dislocation, while point defects contribute less to dechanneling [55, 62, 65].

We succeeded in manufacturing and characterizing two Si crystals with an optimized design for extraction of the SPS or LHC beam. In particular, both interferometric and X-ray measurements agreed in showing a homogeneous curvature of the samples. A large bending angle was achieved both for the samples 1 and 2, which means that the anticlastic behaviour persists also for large bending and for $L/h >1$. The Geant4 simulation predicts that a 7 TeV/c proton beam would be deflected by the sample 1 by the channeling effect with a $73.4\%$ efficiency and that a 400 GeV/c proton beam would be deflected by the sample 2 with a $66.0\%$ efficiency. While sample 1 can be used for beam extraction at LHC, sample 2 could be used for efficient extraction from the SPS [22].

The possibility of obtaining compact crystal deflectors with innovative crystal design has been accomplished and this developed technology could be suitable for a crystal-based extraction in the LHC or in future hadron colliders, such as FCC.
